# EI24, as a Component of Autophagy, Is Involved in Pancreatic Cell Proliferation

**DOI:** 10.3389/fonc.2019.00652

**Published:** 2019-07-23

**Authors:** Mihwa Hwang, Dong Wha Jun, Eun Hye Kang, Kyong-Ah Yoon, Heesun Cheong, Yun-Hee Kim, Chang-Hun Lee, Sunshin Kim

**Affiliations:** ^1^National Cancer Center, Research Institute, Goyang-si, South Korea; ^2^College of Veterinary Medicine, Konkuk University, Seoul, South Korea

**Keywords:** EI24, autophagy, pancreatic cancer cells, tumor promoter, tumor suppressor

## Abstract

Autophagy is a highly conserved cellular process in which cytoplasmic materials are degraded and recycled as energy sources when nutrient supplies are lacking. Established tumor cells require autophagy for cell growth and tumor promotion. In particular, the survival of pancreatic tumor cells appears to be strongly dependent on autophagy, referred to as autophagy addiction. This dependency of pancreatic tumor cells on autophagy may be a candidate target for pancreatic tumor therapy. EI24 (etoposide-induced gene 2.4 kb; PIG8, p53-induced gene 8) acts as a tumor suppressor, inhibiting cell growth and inducing apoptosis in breast, cervical, and prostate cancer cells. However, recent papers have reported that EI24 is an essential component of the autophagy pathway. This newly discovered role of EI24 as a component of autophagy may act as a tumor promoter, which is contradictory to its known role as a tumor suppressor. We investigated the role of EI24 as a component of autophagy in pancreatic tumor cell proliferation. Here, we demonstrated that knockdown of EI24 using siRNA in pancreatic tumor cells led to impaired autophagy at a late step (increase in LC3-II and accumulation of p62 and autolysosomes). EI24 deficiency in pancreatic tumor cell lines inhibited cell proliferation. We confirmed that loss of EI24 inhibited pancreatic cell proliferation using the CRISPR-Cas9 system. However, loss of EI24 in other cell lines did not affect cell proliferation. Taken together, our results suggest that EI24 acts as a tumor promoter in pancreatic tumor cells, and studying the role of EI24 in reference to its cellular context may lead to a useful therapeutic target.

## Introduction

Macroautophagy (hereafter referred to as autophagy) is an evolutionarily conserved process in which an autophagosome engulfs organelles and cytoplasmic materials and delivers them to the lysosome for degradation. In this process, autophagy supplies energy to cells under starvation conditions and also removes harmful or damaged proteins and organelles (such as mitochondria, endoplasmic reticulum, peroxisomes, and lysosomes), which is essential for cell survival and maintaining cellular homeostasis. Recently, involvement of autophagy in human diseases (such as neuronal diseases, metabolic disorders, and cancer) has been reported. The physiological role of autophagy in tumor biology may be to help established tumor cells endure harsh conditions (starvation, growth factor deprivation, and hypoxic stress), promoting tumor cell survival and eventually tumor expansion (1–3). Furthermore, autophagy has been reported to promote proliferation of Ras-driven tumor cells *in vivo* and *in vitro* (4, 5). In particular, pancreatic tumor cells were revealed to depend highly on autophagy for cell growth (6). This autophagy addiction of pancreatic cancer, which is one of the deadliest cancers, is an emerging target for pancreatic cancer therapy.

*EI24* was first identified as an etoposide-inducible p53-dependent gene (7). EI24 expression was reported to play roles in inhibition of cell growth and induction of apoptosis in fibroblasts (8). The *EI24* gene is located on human chromosome 11q23, which is one of the most frequently deleted chromosomal regions in solid tumors. Alteration (point mutation or deletion mutation) and inactivation of *EI24* are correlated with malignancy and invasiveness in breast and cervical cancers (9, 10), and loss of *EI24* in fibroblast and breast tumor cell lines suppressed etoposide-induced apoptosis (11). Decreased EI24 expression is associated with breast tumor invasiveness (12, 13). Based on these findings, EI24 may function as a tumor suppressor gene in breast and cervical cancers. However, a new role of EI24, as an essential component of autophagy, has been identified recently. Detail mechanism has been remained to clarify, however loss of EI24 leads to impaired autophagic flux. EI24 deficiency results in accumulation of LC3, p62, autolysosome, and ubiquitin-positive inclusion, indicating that EI24 is required for degradation step after autolysosome formation (14, 15). Therefore, the function of EI24 as an essential component of autophagy conflicts with its known tumor suppressor role, because autophagy promotes tumor cell survival. In particular, pancreatic tumor cells are highly dependent on autophagy for survival. Thus, the net function of EI24 in pancreatic tumor cells is not clear due to its known tumor suppressor and possible tumor promoter functions. In addition, the role of EI24 as an autophagy factor in pancreatic tumor cells has not yet been clarified. In this study, we examined the autophagic process and cell proliferation of EI24-deficient pancreatic tumor cells and compared the results with other organ-derived cells whose growth is not expected to be affected by EI24 deficiency. We found that loss of EI24 led to impaired autophagy and inhibited cell growth in pancreatic cell lines, but not in other cell lines.

## Materials and Methods

### Cell Culture and siRNA Transfection

MIA PaCa-2, Panc-1, and HCT116 cell lines were obtained from ATCC (Manassas, VA, USA) and HeLa and U2OS cells from KCLB (Seoul, Korea). MIA PaCa-2, Panc-1, HeLa, and U2OS cells were cultured in Dulbecco's modified Eagle medium (DMEM; Corning, Manassas, VA, USA) supplemented with 10% fetal bovine serum (FBS; Corning) at 37°C under a humidified atmosphere with 5% CO_2_. HCT116 cells were maintained in McCoy medium (Corning) supplemented with 10% FBS. All siRNAs targeting EI24 (Hs_EI24_4, Hs_EI24_6, Hs_EI24_7, Hs_EI24_8, and Hs_EI24_9) and ATG5 (Hs_APG5L_6) as well as the negative control siRNA were purchased from QIAGEN (Foster city, CA, USA). Cells were transfected with siRNA (0.1 nmol/10^6^ cells) using Lipofectamine 2000 reagent (Invitrogen, Carlsbad, CA, USA) following the manufacturer's instructions.

### Reverse-Transcription Polymerase Chain Reaction (RT-PCR)

Total RNA from siRNA-transfected cells was extracted using TRIzol reagent (Invitrogen) and then used for cDNA synthesis using the SuperScript™ III First-Strand kit (Invitrogen). PCR was performed using AccuPower™ PCR premix (Bioneer, Daejeon, Korea) with the following primer sets: forward 5′-ATAGAGCGGAAGCAAGAGAGT−3′ and reverse 5′-GCTGTTACCGACTGAAGCACA-3′ for EI24, forward 5′-AGAAGCTGTTTCGTCCTGTGG-3′ and reverse 5′-AGGTGTTTCCAACATTGGCTC-3′ for ATG5, and forward 5′-ACCCAGAAGACTGTGGATGG-3′ and reverse 5′-TTCTAGACGGCAGGTCAGGT-3′ for GAPDH.

### Western Blotting and Antibodies

Cell lysates were prepared for immunoblotting, which was performed as described previously (16). Antibodies targeting the following proteins were used: EI24 (SAB1100756, Sigma Aldrich [St. Louis, MO, USA]), ATG5 (A0731, Sigma Aldrich), LC3 (#2775, Cell Signaling, [Danvers, MA, USA]), p62 (610833, BD), PARP (#9542, Cell Signaling), and β-actin (SC47778, Santa Cruz Biotechnology, Santa Cruz, CA, USA).

### Immunofluorescence Staining

Cells were fixed for immunofluorescence staining, which was performed as described previously (16). Primary antibodies targeting EI24 (NBP2-13949, Novus, [Littleton, CO, USA]) or LC3 (#2775, Cell Signaling) and Alexa Fluor® 488-conjugated anti-rabbit antibodies (Molecular Probes, Carlsbad, CA, USA) were used. Double immunofluorescence staining was performed sequentially. First, the fixed cells were incubated with 1% bovine serum albumin and 0.02% Triton X-100 in phosphate-buffered saline (PBST–BSA) and anti-LC3 antibody (1:500, #2775, Cell Signaling) overnight at 4°C, followed by Alexa Fluor® 488-conjugated anti-rabbit antibodies (1:500, Molecular Probes) for 1 h at room temperature. Alternatively, the fixed cells were incubated with PBST-BSA and anti-LAMP1 antibody (1:500, ab25630, Abcam [Cambridge, UK]) for 3 h at room temperature, followed by Alexa Fluor® 594-conjugated anti-mouse antibodies (1:500, Molecular Probes) and Hoechst 33342 (10 μg/ml, Molecular Probes) for 10 min. The cells were washed three times with PBST for 10 min after each incubation step. After mounting, the cells were visualized under a confocal microscope (Carl Zeiss Microscopy GmbH, Jena, Germany). Pearson's co-localization coefficients were analyzed by ZEN 2.6 (blue edition) as described (17).

### Clonogenic and Crystal Violet Assays

siRNA-transfected cells were subjected to clonogenic assays as described previously (16). For crystal violet staining, siRNA-transfected cells were seeded at 5 × 10^3^/well in a 12-well plate in medium and incubated for 4 days. The cells were fixed with 10% methanol and 10% acetic acid in PBS for 10 min, rinsed with distilled water, dried, and stained with 1% crystal violet in methanol (Sigma Aldrich).

### DNA Ploidy Assay

siRNA-transfected cells were washed with PBS, fixed with cold 70% ethanol, and stored at −20°C for 16 h or longer. The fixed cells were washed with PBS and then incubated in DNA staining solution (10 μg/ml propidium iodide + 1 μg/ml RNase A; Sigma-Aldrich Co.) for 20 min at room temperature in the dark. After incubation, the samples were analyzed by using FACSVerse (BD Pharmingen, San Jose, CA).

### IncuCyte Cell Proliferation Assay

siRNA-transfected cells were seeded at 1 × 10^3^/well in a 96-well plate, which was placed in the IncuCyte ZOOM™ system (ESSEN BioScience, [Ann Arbor, MI, USA]). After incubation for the indicated times live-cell images were obtained using a 10× objective lens (four images per well) within the instrument, and cell density was analyzed using IncuCyte ZOOM 2016B software.

### Cell Death Detection by ELISA

DNA fragmentation was evaluated using the Cell Death Detection ELISA PLUS kit (Roche, Mannheim, Germany). One day after siRNA transfection, cells were seeded at 5 × 10^3^/well in a 96-well plate, incubated for 2 days, and lysed with 200 μl lysis buffer. Then, DNA fragmentation samples were prepared following the manufacturer's protocol. The absorbance was measured at 405 nm using the Versamax microplate reader (Molecular Devices, [Sunnyvale, CA, USA]).

### Lentiviral Transfection of CRISPR-Cas9 EI24 Guide RNA

CRISPR EI24 guide RNAs (gRNAs) 1 and 2 in the pLentiCRISPR v2 vector were purchased from GenScript (Piscataway, NJ). CRISPR EI24 gRNAs and control gRNA in pMDLg/pRRE, pRSV-Rev, and pMD2G (acquired from Addgene, [Watertown, MA, USA]) were transfected into 293T cells to generate lentiviruses. After 2 days, lentiviruses containing CRISPR EI24 gRNA or control gRNA were infected into MIA PaCa-2 cells, and the cells were treated with puromycin (800 μg/ml) for colony selection.

### Xenografts

Five-week-old female Balb/c nu/nu mice were purchased from Orient Bio (Sungnam, Korea). EI24 gRNA- and control gRNA-MIA PaCa-2 cells (5 × 10^6^/100 μl PBS) were injected subcutaneously into the flanks of mice. Tumor size was measured using a caliper, and tumor volume was calculated on the indicated day using the following equation: volume = (length × width^2^)/2. All mice were maintained under specific pathogen-free conditions at the National Cancer Center Research Institute (NCCRI) Animal Facility. All animal experiments in this study were performed in accordance with the institutional guidelines of the NCCRI Animal Facility, an Association for Assessment and Accreditation of Laboratory Animal Care International-accredited institution. This protocol was reviewed and approved by the Institutional Animal Care and Use Committee (IACUC) of NCCRI (No. NCC-18-410).

### Statistical Analysis

Data are presented as mean values ± SEM. All statistical analyses were performed with GraphPad Prism version 5.03 (GraphPad Software, San Diego, CA). Comparisons between two groups were carried out using Student's t-test. Differences between groups were considered statistically significant at P < 0.05.

## Results

### EI24 Is Involved in Autophagy in Pancreatic Cell Lines

**Figure 1:**
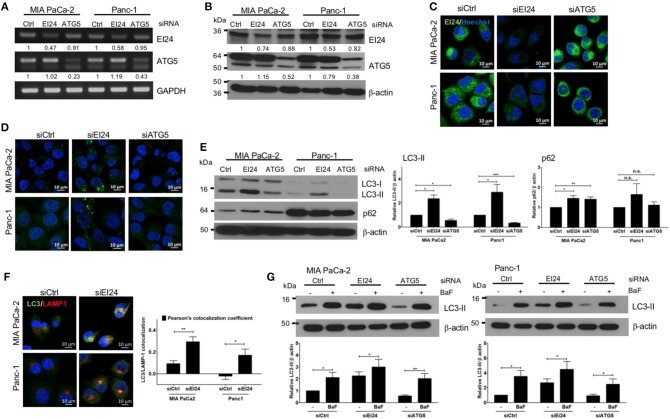
Loss of EI24 expression in pancreatic cancer cells impairs autophagy. MIA PaCa-2 and Panc-1 cells were transfected with 10 nM control siRNA (siCtrl) or siRNA targeting EI24 or ATG5 for 48 h. **(A)** The mRNA levels of EI24, ATG5, and GAPDH were analyzed by reverse-transcription PCR. Values represent the expression of EI24 or ATG5 relative to that of GAPDH (with siCtrl values set to 1). **(B)** The protein levels of EI24 ATG5 and β-actin were analyzed by western blotting. Representative data are shown. Values represent the ratio of the EI24 or ATG5 densitometry value to that of β-actin (with siCtrl values set to 1). **(C)** EI24 protein expression was observed by immunofluorescence staining using an anti-EI24 antibody. **(D)** LC3 puncta (autophagosome) were visualized by immunofluorescence staining using an anti-LC3 antibody. **(E)** Conversion of LC3-I to LC3-II and p62 accumulation were analyzed by western blotting using anti-LC3 and anti-p62 antibodies, respectively. Graphs represent the LC3-II or p62 densitometry value to that of β-actin (with siCtrl values set to 1) from five independent experiments. **(F)** Co-localization of the autophagosome and lysosome in EI24-knockdown MIA PaCa-2 and Panc-1 cells was analyzed by confocal microscopy using the ZEN 2012 program. Immunofluorescence staining with anti-LC3 (to detect autophagosomes; green dye) and anti-LAMP1 (to detect lysosomes; red dye) antibodies. Co-localization of LC3 with LAMP1 was analyzed by ZEN 2.6 (blue edition). The graph shows the Pearson's co-localization coefficient. **(G)** MIA PaCa-2 and Panc-1 cells were treated with 100 nM bafilomycin for 30 min, and their protein extracts were analyzed by western blotting using anti-LC3 and β-actin antibodies. Graphs represent the LC3-II densitometry value to that of β-actin (with siCtrl values set to 1) from three independent experiments. Data in **(E–G)** graphs represent the mean ± standard error of the means (SEM). Comparison were made using Student's *t*-test, ^*^P < 0.05; ^**^P < 0.01; n.s., not significant.

The function of EI24 has been investigated most thoroughly in breast, cervical, and melanoma cells (10, 11, 18). To assess the physiological role of EI24 in pancreatic cells, we conducted transfection experiments using specific siRNAs against EI24 in pancreatic cell lines (MIA PaCa-2 and Panc-1) and analyzed the resulting EI24-deficient pancreatic cells. First, we evaluated a series of siRNAs specific to EI24 (FlexiTube siRNAs #4, #6, #7, #8, and #9 [Qiagen, Foster city, CA, USA]) in MIA PaCa-2 cells and selected siRNA #8 against EI24 (siEI24), which resulted in the most representative phenotype in cell proliferation (Supplementary Figure S1). We measured the mRNA and protein levels of EI24 after transfection of siEI24 #8 into MIA PaCa-2 and Panc-1 cell lines. Decreased mRNA and protein levels of EI24 were confirmed by reverse-transcription polymerase chain reaction (RT-PCR), western blotting, and immunocytochemistry in siEI24-transfected pancreatic cell lines compared with control siRNA (siCtrl)-transfected cells (Figures 1A–C). We also transfected pancreatic cells with siRNA against ATG5 (siATG5) because ATG5 is a key molecule in autophagy; ATG5-deficient pancreatic cells were analyzed alongside EI24-deficient pancreatic cells to verify the role of EI24 in the autophagic process. The mRNA and protein levels of ATG5 were decreased in siATG5-transfected cells, but not in siCtrl- or siEI24-transfected cells (Figures 1A–C and Supplementary Figure S2). Next, we examined whether loss of EI24 in pancreatic cell lines affects autophagy, as impaired autophagic flux in neurons and liver cells of EI24 conditional knockout mice has been reported (15). Based on immunofluorescence staining using an anti-LC3 antibody, the staining of LC3 puncta (autophagosome marker) was increased in siEI24-transfected MIA PaCa-2 and Panc-1 cells, but not in siCtrl-transfected or siATG5-transfected cells (Figure 1D). Western blotting revealed accumulation of LC3-II and p62 in EI24-deficient MIA PaCa-2 and Panc-1 cells (Figure 1E). Conversion of LC3-I to LC3-II is required for autophagosome formation, which indicates the initiation of autophagy. Degradation of p62, a cargo protein in the autolysosome, is the last step of the autophagic process. Thus, accumulation of both LC3-II and p62 represents blockade of autophagic flux (19). To further investigate this mechanism, we transfected RFP-GFP-LC3 plasmid to EI24-deficient MIA PaCa-2 and Panc-1 cells. RFP and GFP double positive LC3 dots (autophagosome), not RFP-positive and GFP-negative LC3 dots (autolysosome) were observed in EI24-deficient MIA PaCa-2 and Panc-1 cells (Supplementary Figure S3). These data supported that EI24 knockdown impaired autophagic flux. Furthermore, immunofluorescence staining and Pearson's co-localization coefficient analysis showed that LC3 (autophagosome marker) and LAMP1 (lysosome marker) were co-localized in EI24-deficient MIA PaCa-2 and Panc-1 cells (Figure 1F), suggesting that loss of EI24 did not affect autolysosome formation. When EI24-deficient cells were treated with bafilomycin A1, which inhibits autophagy at the autolysosomal formation and degradation steps, further LC3-II accumulation was observed (Figure 1G). Taken together, these results indicate that EI24 may be involved in autophagy in pancreatic cell lines at the final stage, after autolysosomal formation. Involvement of EI24 in the last step of the autophagic process was also reported in the model organism *Caenorhabditis elegans* (14). We determined that loss of EI24 inhibited autophagy at the degradation step in the autolysosome of pancreatic cell lines.

### Loss of EI24 Inhibits Pancreatic Cell Growth

**Figure 2:**
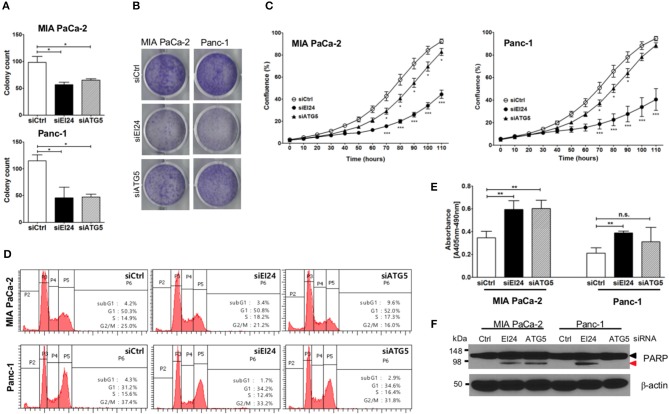
Loss of EI24 expression in pancreatic cancer cells inhibits cell proliferation. MIA PaCa-2 and Panc-1 cells were transfected with 10 nM control siRNA (siCtrl) and siRNA targeting EI24 or ATG5. **(A)** After 24 h of transfection, cells were reseeded into a 6-well plate and incubated for 10 days. Then, the cells were fixed and colonies counted. The values represent means ± SEM (Student's *t*-test, ^*^P < 0.05). **(B)** After 24 h of transfection, cells were seeded into a 6-well plate and incubated for another 7 days. Cells were fixed and stained with crystal violet. **(C)** After 24 h of transfection, cells were seeded into a 96-well plate. Images acquired from the IncuCyte instrument at the indicated times were analyzed using the ZOOM 2016 program. Cell confluency was measured in triplicate wells for each sample. The plotted values represent means ± SEM (Student's *t*-test, ^***^P < 0.001). **(D)** Cells transfected with siRNAs were incubated for 48 h. After incubation, the cells were analyzed by flow cytometry to evaluate DNA content. Representative DNA content profiles from three independent experiments are shown. **(E)** DNA fragmentation in siRNA-transfected MIA PaCa-2 and Panc-1 cell lysates was measured using the Cell Death Detection kit. The *y*-axes in the graphs indicate the extent of DNA fragmentation. Values plotted in the graphs represent means ± SEM. (Student's *t*-test, ^**^P < 0.01; n.s., not significant). **(F)** Proteins extracted from siRNA-transfected cells was analyzed by western blotting using anti-PARP1 and anti-β-actin antibodies. The proform of PARP (116 kDa, black arrow head) and cleaved PARP (85 kDa, red arrow head) are indicated.

Next, we determined whether EI24 deficiency leads to impaired pancreatic cancer cell growth. Because pancreatic cancer cells depend on autophagy for cell survival, loss of the autophagic component EI24 may inhibit pancreatic cancer cell growth. We analyzed cell proliferation using the colony formation, crystal violet, and IncuCyte cell proliferation assays after transfection of siEI24 into MIA PaCa-2 and Panc-1 cells. EI24-deficient pancreatic cells exhibited decreased cell proliferation compared with siCtrl-transfected cells (Figures 2A–C). ATG5-deficient pancreatic cells also showed decreased cell proliferation. Thus, knockdown of autophagy-related genes (such as EI24 and ATG5) inhibited the growth of pancreatic cell lines. EI24 acts as a tumor suppressor gene in breast and cervical cancer cells, but it appears to play a different role in pancreatic cancer cells based on these results. And then we examined whether EI24 overexpression affects to cell proliferation. We analyzed cell proliferation using IncuCyte assay after transfection of pcDNA3-EI24 plasmid into MIA PaCa-2 and Panc-1 cells. EI24-overexpressed MIA PaCa-2 cells exhibited increased cell proliferation compared to pcDNA3-transfected cells. But EI24-overexpressed Panc-1 cells did not showed alternation in cell proliferation (Supplementary Figure S4). Besides we did not observed alteration of LC3-II and p62 in EI24-overexpressed MIA PaCa-2 and Panc-1 cells. Taken together, EI24 overexpression in pancreatic cancer cells did not activate autophagy process and then did not raise cell proliferation.

Next, we determined the mechanism of siEI24-induced cell growth inhibition resulted from cell cycle arrest. Based on a DNA ploidy assay using propidium iodide staining and flow cytometric analysis, the cell cycle was not altered in EI24-deficient pancreatic cell lines (Figure 2D). Next, we investigated whether siEI24-induced cell growth inhibition resulted from cell death. We measured DNA fragmentation, which is a marker of apoptosis, using an enzyme-linked immunosorbent assay (ELISA) kit for DNA fragmentation. DNA fragmentation was slightly elevated in EI24-deficient MIA PaCa-2 and Panc-1 cells (Figure 2E). Furthermore, we assessed cleavage of poly ADP-ribose polymerase (PARP), another marker of apoptosis. Loss of EI24 induced PARP cleavage in pancreatic cells (Figure 2F). DNA fragmentation and PARP cleavage were also observed in ATG5-deficient cells. However, an increase in the sub-G1 peak, another indication of apoptosis, was not observed in EI24-deficient pancreatic cells (Figure 2D). We did not observe recovery of apoptosis after treatment with a pan-caspase inhibitor (Q-VD-Oph) (data not shown). Taken together, these results suggest that apoptosis is involved in siEI24-induced growth inhibition of pancreatic cell lines partially.

### EI24 Deficiency Did Not Affect the Proliferation of Other Cell Lines

**Figure 3:**
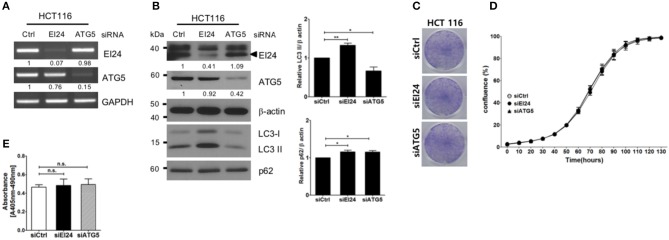
Loss of EI24 expression in HCT116 cells impairs autophagy but not cell proliferation. HCT116 cells were transfected with 10 nM control siRNA (siCtrl) or siRNA targeting EI24 or ATG5. **(A)** The mRNA levels of EI24, ATG5, and GAPDH were analyzed by reverse-transcription PCR. **(B)** The protein levels of EI24 ATG5 and β-actin were analyzed by western blotting. Representative data are shown. Values represent the ratio of the EI24 or ATG5 densitometry value to that of β-actin (with siCtrl values set to 1). Conversion of LC3-I to LC3-II and p62 accumulation were analyzed by western blotting using anti-LC3 and anti-p62 antibodies, respectively. Graphs represent mean ± SEM of the LC3-II and p62 densitometry value to that of β-actin (with siCtrl values set to 1) from three independent experiments. Comparison were made using Student's *t*-test, ^*^P < 0.05; ^**^P < 0.01. **(C)** After 24 h of transfection, cells were seeded into a 6-well plate and incubated for another 7 days. Cells were fixed and stained with crystal violet. **(D)** After 24 h of transfection, cells were seeded into a 96-well plate. Images acquired from the IncuCyte instrument at the indicated times were analyzed using the ZOOM 2016 program. Cell confluency was measured in triplicate wells for each sample. The plotted values represent means ± SEM. **(E)** Protein extracts from siRNA-transfected cells were analyzed for DNA fragmentation. The *y*-axes of the graphs indicate the extent of DNA fragmentation. Values plotted in the graphs represent means ± SEM (Student's *t*-test, n.s., not significant).

Next, we examined whether loss of EI24 affects the growth of other cell lines. First, we confirmed a decreased EI24 mRNA level in HCT116 cells after siEI24 transfection using RT-PCR (Figure 3A). Next, we measured autophagic flux in HCT116 cells after siEI24 transfection. Accumulation of LC3-II and p62 levels was observed in EI24-knockdown HCT116 cells, indicating that loss of EI24 expression leads to impaired autophagic flux in other cell lines as well (Figure 3B). Whereas, the proliferation of pancreatic cells was inhibited by EI24 deficiency, that of HCT116 cells was not, according to crystal violet and IncuCyte assays (Figures 3C,D). DNA fragmentation was not altered in EI24-deficient HCT116 cells (Figure 3E). We observed that EI24 deficiency in other cell lines, including HeLa and U2OS cells, did not affect cell proliferation (Supplementary Figure S5). Collectively, these findings indicate that EI24 deficiency may inhibit the growth of pancreatic cells that require autophagy for their survival, but not other cell lines.

### Loss of EI24 Using the CRISPR-Cas9 System Inhibits Pancreatic Cell Proliferation

**Figure 4:**
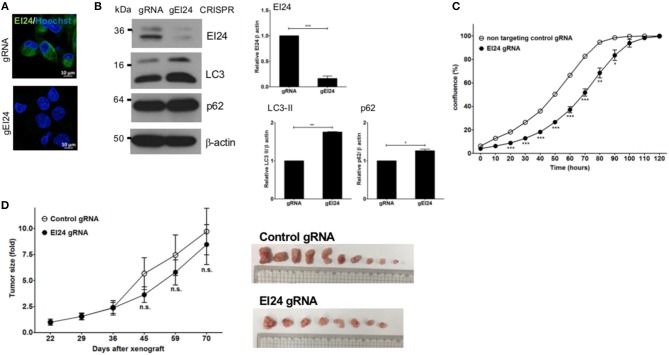
Loss of EI24 expression using CRISPR-Cas9 in MIA PaCa-2 cells decreased cell proliferation. MIA PaCa-2 cells were transfected with CRISPR-Cas9 control (gRNA) and EI24 gRNA (gEI24) using a lentiviral system. **(A)** After 48 h of incubation, EI24 protein expression was observed by immunofluorescence staining using an anti-EI24 antibody. **(B)** After 48 h of incubation, the EI24 protein level was observed by western blotting using an anti-EI24 antibody. Conversion of LC3-I to LC3-II and p62 accumulation were analyzed by western blotting using anti-LC3 and anti-p62 antibodies, respectively. Graphs represent the mean ± SEM of EI24, LC3-II, and p62 densitometry value to that of β-actin (with gRNA values set to 1) from three independent experiments. Comparison were made using Student's *t*-test, ^*^P < 0.05; ^**^P < 0.01; ^***^P < 0.001. **(C)** After 24 h of incubation, cells were seeded into a 96-well plate (1,000 cells/well). Images acquired by the IncuCyte instrument at the indicated times were analyzed using the ZOOM 2016 program. Confluency was measured in triplicate wells for each sample. Values represent the means ± SEM (Student's *t*-test, ^*^P < 0.05; ^**^P < 0.01; ^***^P < 0.001). **(D)** Control and EI24 gRNA-transfected cells (5 × 10^6^) were injected into both flanks of Balb/c nude mice. Tumor volume was measured on the indicated days. The *y*-axes of these graphs represent the fold change in tumor size relative to the initial tumor size. Values represent means ± SEM. (Student's *t*-test, n.s., not significant, control gRNA mice, *n* = 5; EI24 gRNA mice, *n* = 4).

We also assessed induced EI24 deficiency in pancreatic cells using the CRISPR-Cas9 knockdown system. We introduced EI24 or non-target CRISPR guide RNA into MIA PaCa-2 cells via lentiviruses and measured the EI24 expression levels in selected clones following puromycin treatment. We chose a representative EI24-knockdown MIA PaCa-2 cell clone for detailed assessments, including a xenograft experiment. Using immunocytochemistry and western blotting, the EI24 protein level was decreased in EI24 gRNA-MIA PaCa-2 cells compared with control gRNA cells (Figure 4A). As both accumulation of LC3-II and p62 in EI24 gRNA-MIA PaCa-2 cells was detected by western blotting, we confirmed that loss of EI24 using the CRISPR-Cas9 system led to impaired autophagic flux (Figure 4B). Next, we measured pancreatic cell proliferation in these EI24 gRNA-MIA PaCa-2 cells using the IncuCyte proliferation assay, which showed reduced proliferation, in accordance with the siRNA results (Figure 4C). Then, we used a xenograft model to assess whether EI24 deficiency inhibits pancreatic cell proliferation *in vivo*. EI24 and control gRNA-MIA PaCa-2 cells were injected subcutaneously into Balb/c nude mice, and the resulting tumor size was measured. Tumors developed slowly in mice injected with EI24-deficient cells compared with control gRNA cells, but this difference was not statistically significant (Figure 4D).

## Discussion

EI24 has been identified as an etoposide-inducible transcript and as a tumor suppressor inducing apoptosis in breast, cervical and prostate cancer cells. On the other hand, EI24 has been identified as a component of autophagy, indicating that EI24 plays a different role under autophagy-dependent conditions. In this study, we clarified that loss of EI24 impaired autophagy and inhibited proliferation in pancreatic cancer cells, which are dependent on autophagy. We suggest that EI24, despite being a tumor suppressor, also acts as a tumor promoter in pancreatic cancer cells. This is not the first report that EI24 acts as a tumor promoter. Devkota et al. reported that DMBA/TPA-induced skin carcinogenesis was attenuated in EI24 heterozygous mice compared with wild-type mice (20). They suggested that EI24 acts as a tumor suppressor in skin cancer. Additionally, it has been reported that EI24 is involved in the survival and growth of non-tumor cells such as pancreatic β-cells and *Dictyostelium discoideum* (21, 22). EI24 has been reported as a tumor suppressor even in pancreatic cancer cells, which is in conflict with our observations (23). In that study, knockdown of EI24 in CaPan2 cells did not inhibit cell proliferation or LC3-I to LC3-II conversion in CaPan2 cells. We cannot easily explain the discrepant results achieved by EI24 knockdown in pancreatic cancer cells, but they may be related to the differences in cell lines or delivery systems. In addition, p53 status of cells might be a key molecule for cancer progression, which is mutated in MIA PaCa-2 and Panc-1, but not in CaPan2. Further detailed experiments to investigate these differences will be needed. Recently, a novel function of EI24 in inhibiting nuclear import by binding IMPβ1 and IMPα2 was reported (24). And other group suggested that EI24 is essential for crosstalk between autophagy and the ubiquitin–proteasome system via degradation of RING-domain E3 ligase (25). The various binding partners and functions of EI24 might cause different results depending on the cellular and environmental conditions. In this study, EI24 deficiency did not affect cell proliferation in HCT116, colon cancer, HeLa, or U2OS cells, unlike in pancreatic cancer cells. These data supported the possibility of multiple roles of EI24.

We used microarray analysis (Agilent Human whole-genome 44K) to compare mRNA expression levels between EI24-proficient and -deficient pancreatic cells and analyzed their molecular profiles to clarify the mechanism of proliferation inhibition in EI24-deficient cells. Upregulated (65 genes) and downregulated (89 genes) genes were detected in both MIA PaCa-2 and Panc-1 cells after EI24 knockdown (Supplementary File 1). Among these genes, five upregulated genes were related to apoptosis and six downregulated genes to cell proliferation. However, no obvious gene profile alterations explaining the inhibitory mechanism of EI24-deficient pancreatic cell proliferation was identified.

The autophagy dependency of pancreatic cancer cells may lead to inhibition of cell proliferation after loss of autophagic components, such as EI24 and ATG5. However, the proliferation of cancer cell lines such as HCT116 colon cancer, HeLa cervical cancer and U2OS osteosarcoma cells were not affected by loss of these autophagic components. EI24 acts as a tumor suppressor in cervical, breast, and prostate cancers. However, in this study, EI24 appeared to play a different role in pancreatic cancer cells, suggesting varying roles depending on the cellular context.

In conclusion, we suggest that EI24, as a component of autophagy, may promote pancreatic cancer cell growth.

## Data Availability

All datasets generated for this study are included in the manuscript and/or the Supplementary Files.

## Ethics Statement

All animal experiments in this study were performed in accordance with the institutional guidelines of the National Cancer Center Animal Facility, an Association for Assessment and Accreditation of Laboratory Animal Care International-accredited institution.

## Author Contributions

Conceptualization: SK. Methodology: SK and MH. Validation: MH, DJ, and EK. Formal analysis: SK and MH. Investigation: SK. Resources: K-AY and Y-HK. Writing-original draft preparation: SK and MH. Writing-review and editing: SK, K-AY, HC, Y-HK, and C-HL.

### Conflict of Interest Statement

The authors declare that the research was conducted in the absence of any commercial or financial relationships that could be construed as a potential conflict of interest.

## Supplementary Material

Supplementary Figure S1 Selection of siRNA against EI24 MIA PaCa-2 cells were transfected with 10 nM siRNAs against control (Ctrl) or EI24 (#4, #6, #7, #8 and #9). **(A)** After 48 h of transfection, the mRNA level of EI24 and GAPDH were analyzed by reverse-transcript PCR. **(B)** The protein level of b-actin, EI24 and LC3 were analyzed by western blotting. **(C)** After 24 h of transfection, cells were reseeded into 96-well plate as triplicate and analyzed cell confluency using IncuCyte instrument and ZEN2016 program.

Supplementary Figure S2 ATG5 protein expression level after siATG5 transfection MIA PaCa-2 and Panc-1 cells were transfected with 10 nM siRNA against control (Ctrl), EI24 and ATG5 for 48 h. ATG5 protein expression was observed by immunofluorescence staining using anti-ATG5 antibody. (Green: ATG5 and Blue (Hoechst 33342): nucleus).

Supplementary Figure S3 RFP-GFP-LC3 transfection to EI24-knockdowned cells MIA PaCa-2 and Panc-1 cells were transfected with 10 nM siRNA against control (Ctrl) and EI24. After 48 h the first round of transfection, cells were retransfected with 10 nM siCtrl or siEI24 in combination with 1 μg of pcDNA3-RFP-GFP-LC3 plasmid. After 24 h of retransfection, cells were fixed with 3.7% formalin, stained with Hoechst33342 and observed with a confocal microscope. (Blue (Hoechst 33342): nucleus, Yellow (RFP(+)-GFP(+)-LC3): autophagosome, Red (RFP(+)-GFP(-)-LC3): autolysosome).

Supplementary Figure S4 Overexpression of EI24 in pancreatic cancer cells **(A)** MIA PaCa-2 and Panc-1 cells (1 X 105 cells) were transfected with 0.5 μg of pcDNA3 and pcDNA3-EI24. After 24 hr transfection, cells were reseeded on 96 well plate as triplicate and analyzed confluency at the indicated time using IncuCyte instrument and ZEN2016 program. **(B)** The protein level of b-actin, EI24-flag, LC3 and p62 were analyzed by western blotting.

Supplementary Figure S5 Loss of EI24 in cell proliferation of HeLa and U2OS 886 HeLa and U2OS cells were transfected with 10 nM siRNA against control (Ctrl), EI24 and ATG5. **(A)** After 24 h of transfection, cells were reseeded into 96 well plate as triplicate and analyzed confluency at the indicated time using IncuCyte instrument and ZEN2016 program. **(B)** The protein level of b-actin, EI24, ATG5, LC3 and p62 were analyzed by western blotting.

Supplementary File 1 EI24 knockdown in pancreatic cancer cells.
