# Supplementary material for: EI24, as a Component of Autophagy, Is Involved in Pancreatic Cell Proliferation
Source: Front Oncol. 2019 Jul 23;9:652. doi: 10.3389/fonc.2019.00652 (PMC6664870; doi:10.3389/fonc.2019.00652)
Supplement: Supplementary Figure S1 — Selection of siRNA against EI24 MIA PaCa-2 cells were transfected with 10 nM siRNAs against control (Ctrl) or EI24 (#4, #6, #7, #8 and #9). (A) After 48 h of transfection, the mRNA level of EI24 and GAPDH were analyzed by reverse-transcript PCR. (B) The protein level of b-actin, EI24 and LC3 were analyzed by western blotting. (C) After 24 h of transfection, cells were reseeded into 96-well plate as triplicate and analyzed cell confluency using IncuCyte instrument and ZEN2016 program. [file Presentation_1.pptx]

## Slide 1
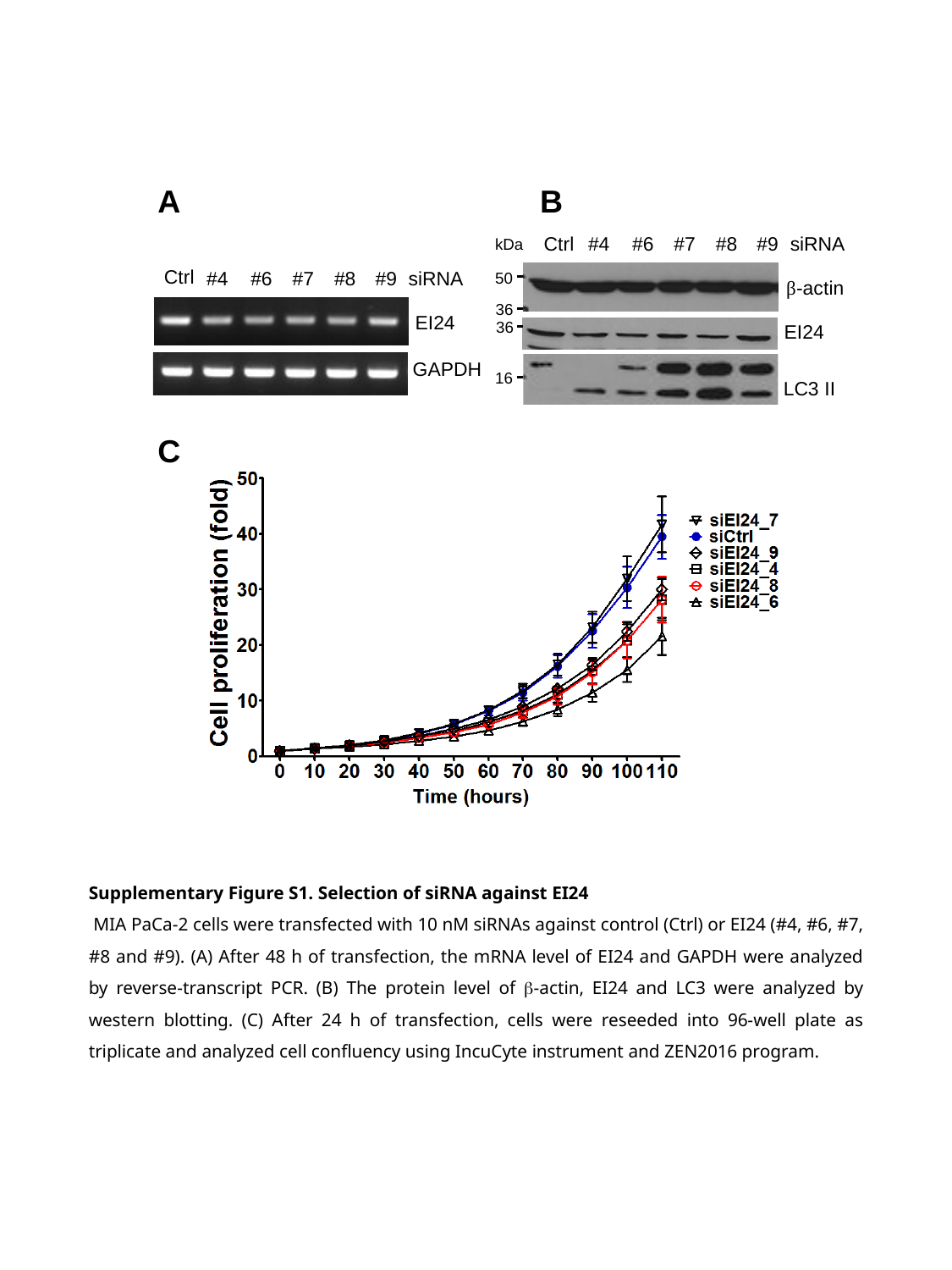

B
Ctrl
#4
#6
#7
#8
#9
siRNA
kDa
50
36
β-actin
36
EI24
16
LC3 II
A
Ctrl
#4
#6
#7
#8
#9
siRNA
EI24
GAPDH
C
Supplementary Figure S1. Selection of siRNA against EI24
 MIA PaCa-2 cells were transfected with 10 nM siRNAs against control (Ctrl) or EI24 (#4, #6, #7, #8 and #9). (A) After 48 h of transfection, the mRNA level of EI24 and GAPDH were analyzed by reverse-transcript PCR. (B) The protein level of β-actin, EI24 and LC3 were analyzed by western blotting. (C) After 24 h of transfection, cells were reseeded into 96-well plate as triplicate and analyzed cell confluency using IncuCyte instrument and ZEN2016 program.

## Slide 2
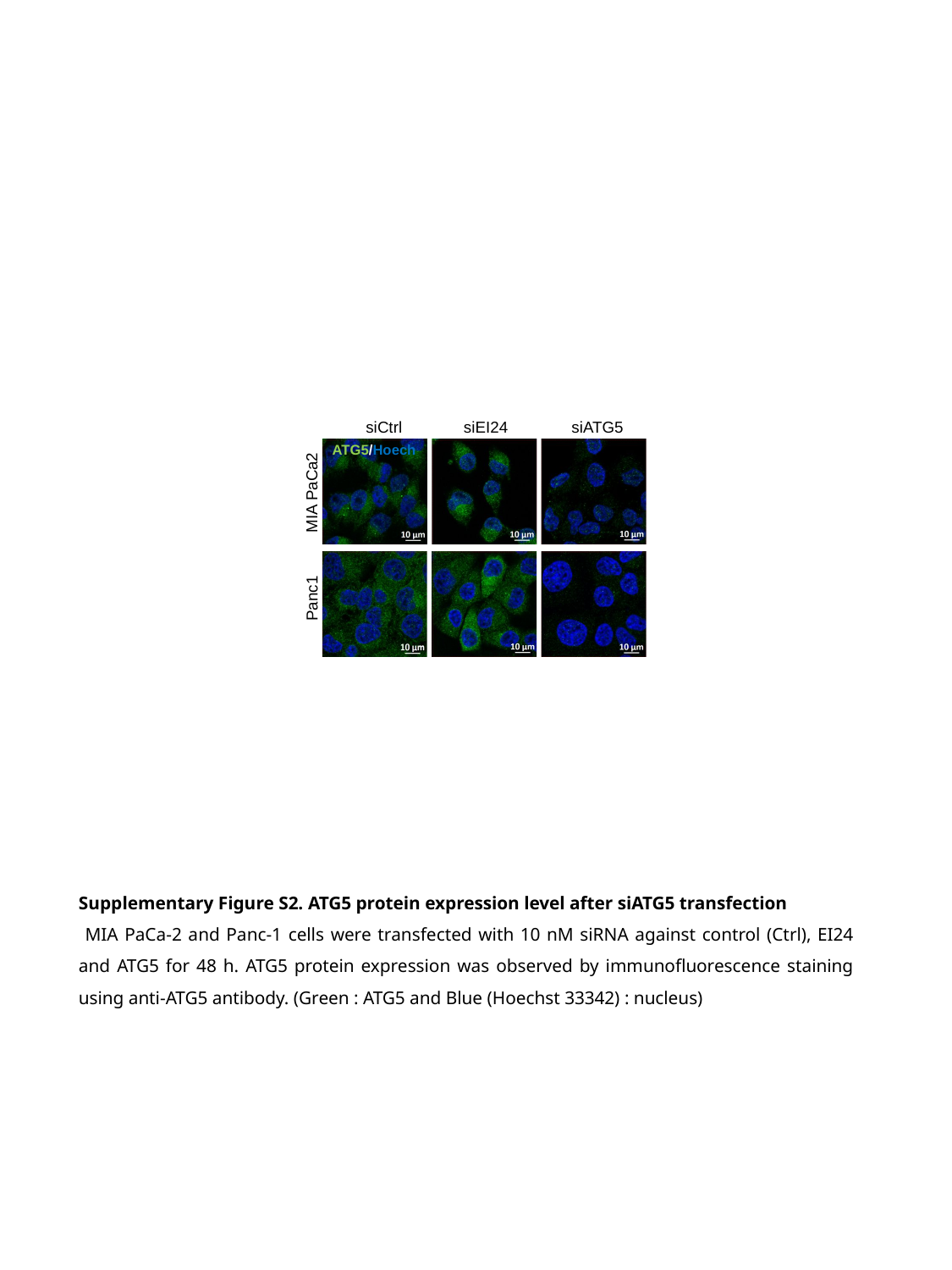

siCtrl
siEI24
siATG5
MIA PaCa2
Panc1
ATG5/Hoech
Supplementary Figure S2. ATG5 protein expression level after siATG5 transfection
 MIA PaCa-2 and Panc-1 cells were transfected with 10 nM siRNA against control (Ctrl), EI24 and ATG5 for 48 h. ATG5 protein expression was observed by immunofluorescence staining using anti-ATG5 antibody. (Green : ATG5 and Blue (Hoechst 33342) : nucleus)

## Slide 3
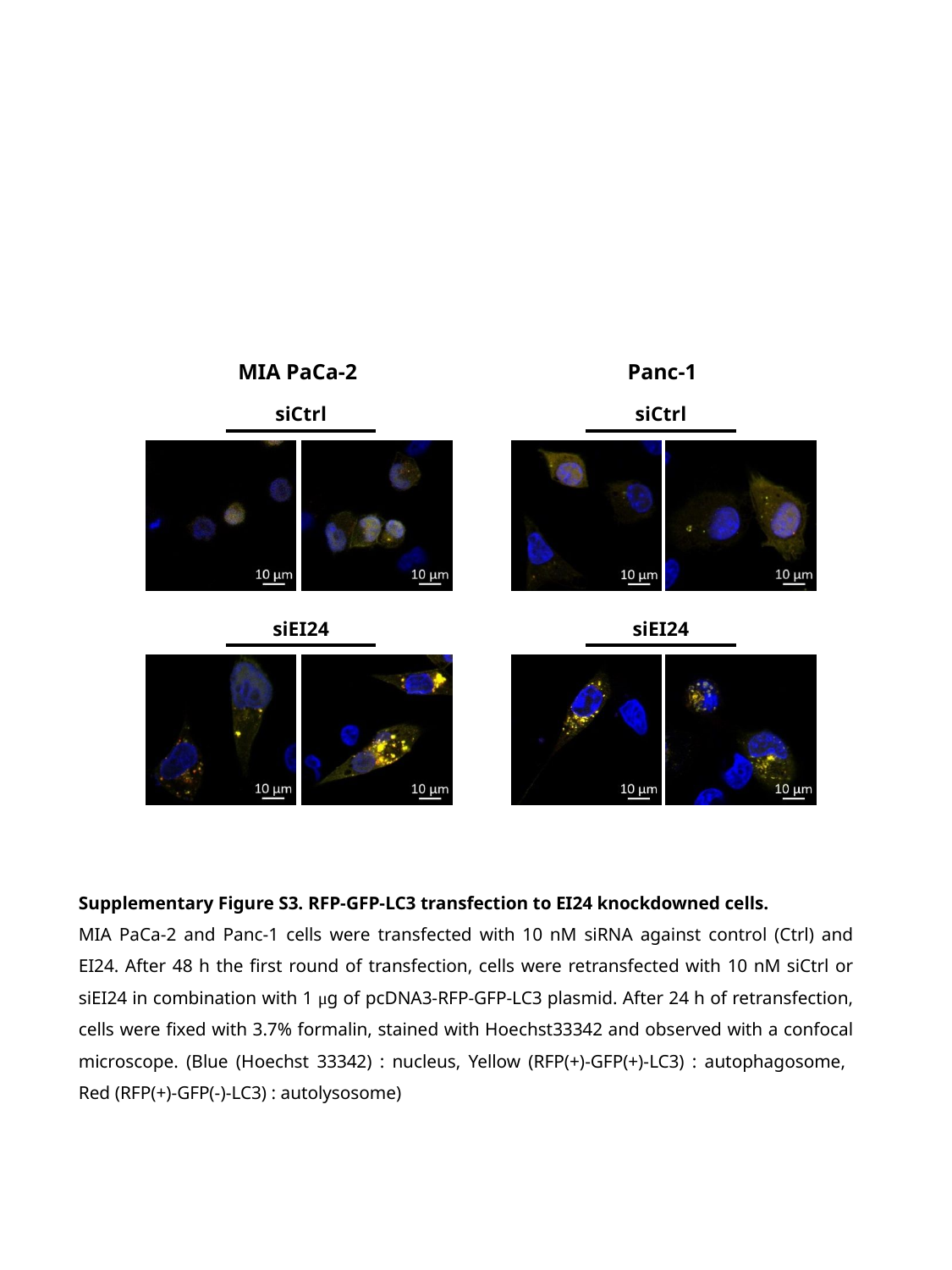

MIA PaCa-2
Panc-1
siCtrl
siCtrl
siEI24
siEI24
Supplementary Figure S3. RFP-GFP-LC3 transfection to EI24 knockdowned cells.
MIA PaCa-2 and Panc-1 cells were transfected with 10 nM siRNA against control (Ctrl) and EI24. After 48 h the first round of transfection, cells were retransfected with 10 nM siCtrl or siEI24 in combination with 1 μg of pcDNA3-RFP-GFP-LC3 plasmid. After 24 h of retransfection, cells were fixed with 3.7% formalin, stained with Hoechst33342 and observed with a confocal microscope. (Blue (Hoechst 33342) : nucleus, Yellow (RFP(+)-GFP(+)-LC3) : autophagosome, Red (RFP(+)-GFP(-)-LC3) : autolysosome)

## Slide 4
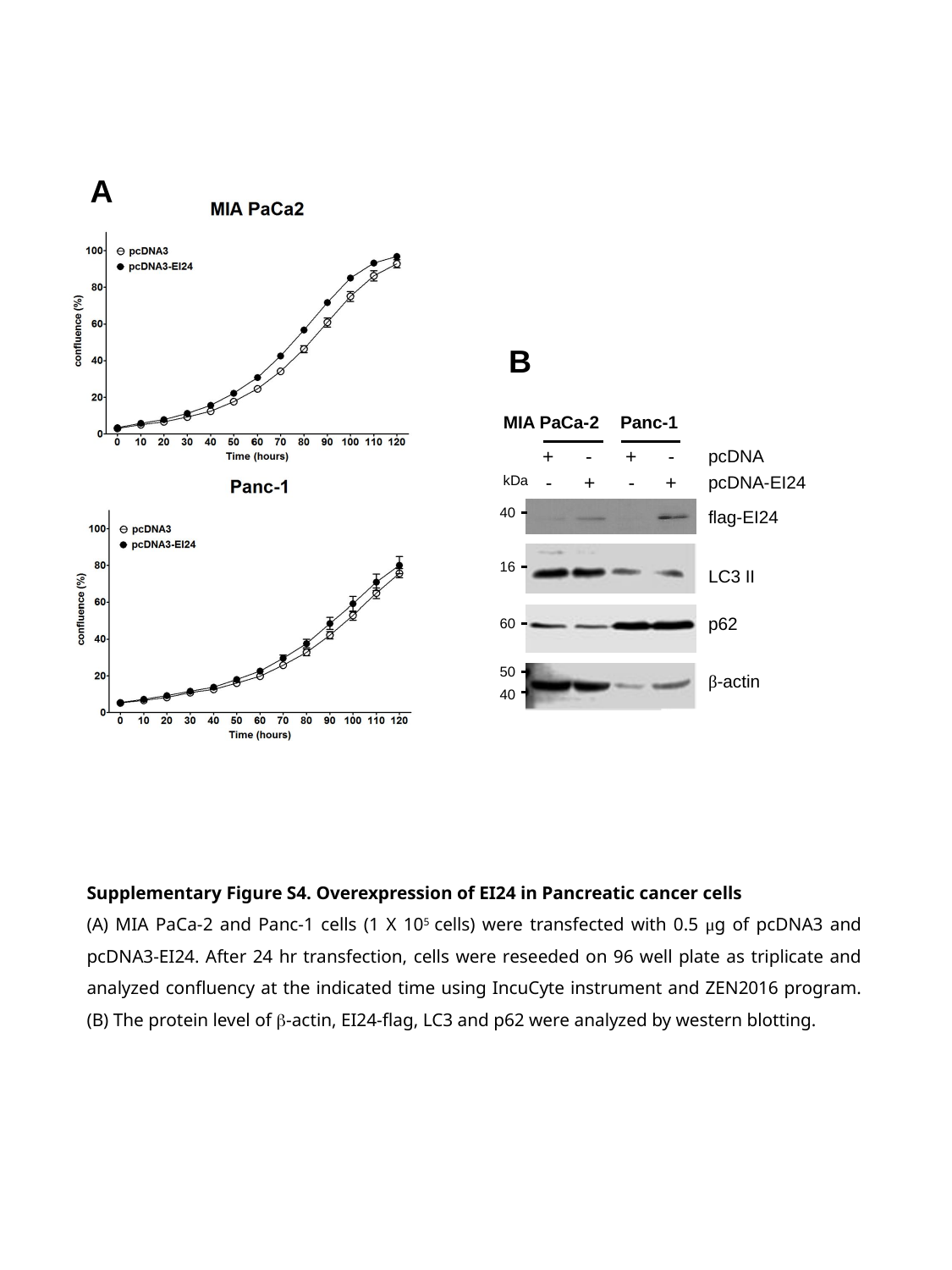

A
B
MIA PaCa-2
Panc-1
+
-
+
-
pcDNA
-
+
-
+
 kDa
pcDNA-EI24
40
flag-EI24
16
LC3 II
p62
60
50
β-actin
40
Supplementary Figure S4. Overexpression of EI24 in Pancreatic cancer cells
(A) MIA PaCa-2 and Panc-1 cells (1 X 105 cells) were transfected with 0.5 μg of pcDNA3 and pcDNA3-EI24. After 24 hr transfection, cells were reseeded on 96 well plate as triplicate and analyzed confluency at the indicated time using IncuCyte instrument and ZEN2016 program. (B) The protein level of β-actin, EI24-flag, LC3 and p62 were analyzed by western blotting.

## Slide 5
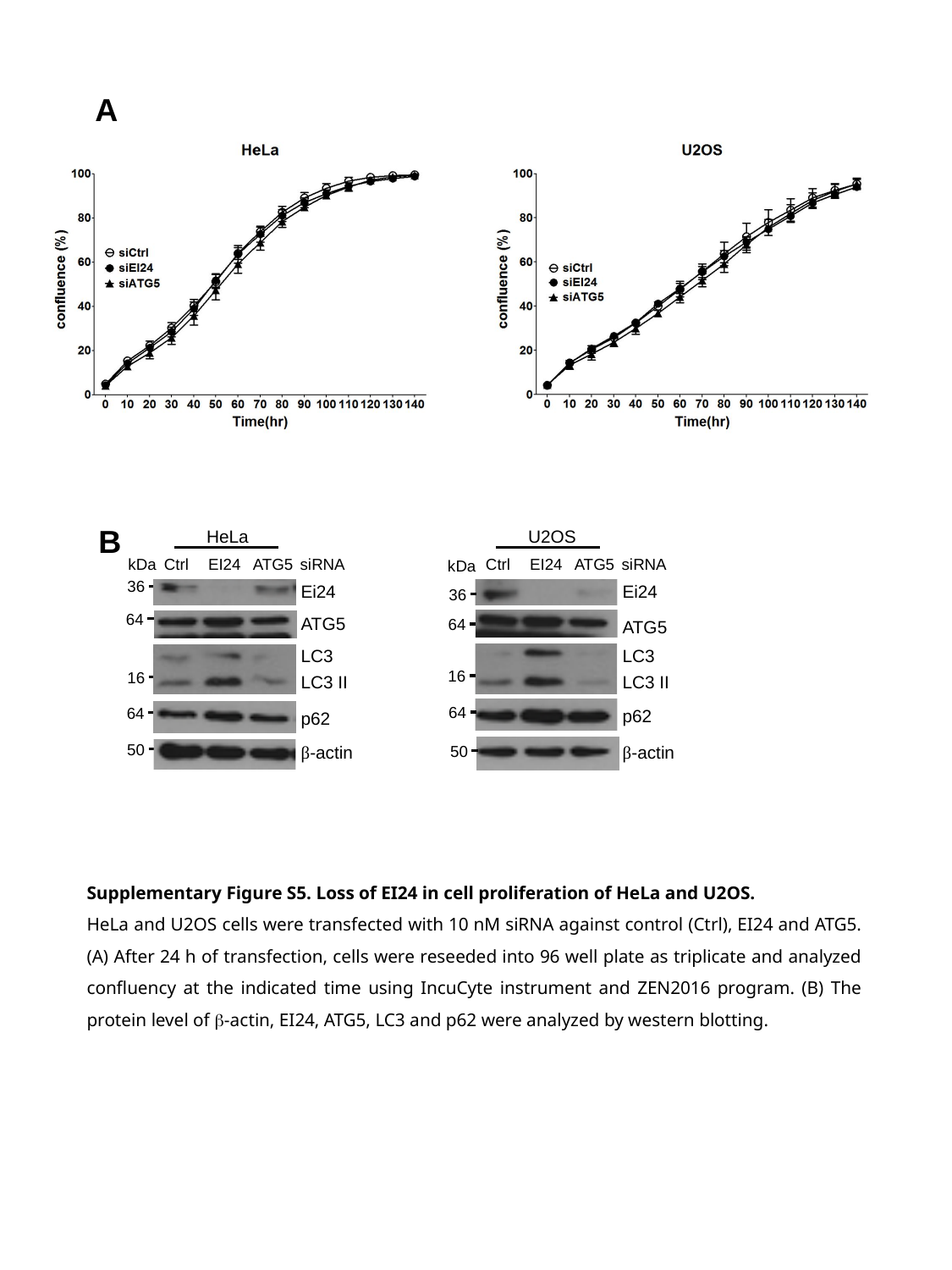

A
B
HeLa
Ctrl
EI24
ATG5
siRNA
Ei24
ATG5
LC3
LC3 II
p62
β-actin
kDa
36
64
16
64
50
U2OS
Ctrl
EI24
ATG5
siRNA
Ei24
ATG5
LC3
LC3 II
p62
β-actin
 kDa
36
64
16
64
50
Supplementary Figure S5. Loss of EI24 in cell proliferation of HeLa and U2OS.
HeLa and U2OS cells were transfected with 10 nM siRNA against control (Ctrl), EI24 and ATG5. (A) After 24 h of transfection, cells were reseeded into 96 well plate as triplicate and analyzed confluency at the indicated time using IncuCyte instrument and ZEN2016 program. (B) The protein level of β-actin, EI24, ATG5, LC3 and p62 were analyzed by western blotting.
